# On Connectivity and Energy Efficiency for Sleeping-Schedule-Based Wireless Sensor Networks

**DOI:** 10.3390/s19092126

**Published:** 2019-05-08

**Authors:** Lijun Wang, Jia Yan, Tao Han, Dexiang Deng

**Affiliations:** 1 Electronic Information School, Wuhan University, Wuhan 430072, China; wanglijun@whu.edu.cn (L.W.); ddx@whu.edu.cn (D.D.); 2 Faculty of Information Science and Technology, Wenhua College, Wuhan 430074, China; 3 School of Electronic Information and Communications, Huazhong University of Science and Technology, Wuhan 430074, China; hantao@hust.edu.cn

**Keywords:** wireless sensor networks, connectivity, energy efficiency, power allocation

## Abstract

Based on the connectivity and energy consumption problems in wireless sensor networks, this paper proposes a kind of new network algorithm called the connectivity and energy efficiency (CEE) algorithm to guarantee the connectivity and connectivity probability, and also to reduce the network energy consumption as much as possible. Under the premise that all sensors can communicate with each other in a specific communication radius, we obtained the relationship among the connectivity, the number of sensor nodes, and the communication radius because of the theory of probability and statistics. The innovation of the paper is to maximize the network connectivity and connectivity probability, by choosing which types of sleeping nodes to wake up. According to the node’s residual energy and the relative value of distance, the algorithm reduces the energy consumption of the whole network as much as possible, and wakes up the number of neighbor nodes as little as possible, to improve the service life of the whole network. Simulation results show that this algorithm combines the connectivity and the energy efficiency, provides a useful reference value for the normal operation of the sensors networks.

## 1. Introduction

With the rapid development and maturation of wireless communication technology, micro-motor system low-power consumption and highly-integrated digital devices enable the realization of low-cost, low-power, and small-volume sensor nodes. Such sensor nodes, together with the wireless communication networks, form self-organizing wireless sensor networks (WSNs) and are widely used in battlefield surveillance, large-scale environmental monitoring, and target tracking in large areas. Sensors are responsible for monitoring the environment, transferring, and processing the data to the WSN base stations. Since the WSN nodes are generally powered by batteries, which are difficult to replace due to the large number and harsh working environment, one of the crucial issues in the construction of a large-scale wireless sensor network is the limited battery life cycle [1]. Low power consumption plays a critical role and the energy efficiency of nodes is one of the most critical design criteria for wireless sensor networks [2,3]. For wireless sensor networks, connectivity is of great significance for wireless network planning, capacity design, topology control, and node power optimization. Therefore, it is of great theoretical and practical significance to ensure the connectivity of WSN and realize its low power consumption at the same time, to minimize the energy consumption of the network and improve the service life of the network.

Percolation is a subject that studies various structures of graphs based on probability analysis. Its central problem is to study the threshold of connectivity of various graphs, which has large application value in social life and scientific research. In recent years, many researchers have conducted research work on ad hoc networks, most of them focusing on network coverage and network routing algorithms. Li et at. [4] analyzed the number of nodes in the large-scale network by using the theory of continuous flow and analyzed the connectivity of the large-scale component. In [5], for wireless sensor networks with randomly-placed nodes as the background, graph theory, and statistics are used as the primary research methods to study the relationship among the number of nodes, the communication radius of nodes, and the network connectivity. The paper established a corresponding mathematical model, carried out a large number of computer simulation experiments, and obtained the mathematical analytic formula between them. Wang et al. [6] joined on the connectivity and coverage together, analyzed the characteristics of WSN with random deployment way, by considering the distribution of sensor nodes in the monitoring area as a Poisson point process. Aiming at the characteristics of random deployment, the paper gave the node density to satisfy the network connectivity requirement in the case of the node sensing range and communication range. According to the analysis results, it proposed a location-independent node self-scheduling connectivity covering algorithm (LISCCA). Agrawal et al. [7] mainly studied the influence of the lognormal shadow wireless transmission model on the connectivity of the wireless network, introduced the connection function to describe the existence of wireless links between nodes, and then abstracted the partial connectivity issues as the seepage problem of special stochastic graph. Finally, the theoretical upper bound of the critical node density was deduced in a partially connected state to solve the problem of heavy maintenance in wireless sensor networks. Chai et al. [8] proposed a networking algorithm with good connectivity performance, which contributed to the energy efficiency optimization of WSNs. Mostafaei et al. [9] also proposed an irregular cellular learning automaton (ICLA)-based algorithm to preserve sensors protection. It performs on average of 50% better than the maximum independent set and minimum connected dominating set algorithms in terms of active node ratio while providing two times reduction in energy consumption. 

However, there are few literatures devoted to the study of wireless sensor network connectivity and networking algorithm from the aspect of probability theory. Based on the predecessors’ research with the connectivity of wireless sensor networks, this paper studies this problem and presents the innovation on the combination of the probability theory and a new network algorithm called connectivity and energy efficiency (CEE) algorithm with the high connectivity. By applying the principles of computer network router to the wireless sensor nodes, aiming at achieving the maximum probability of connectivity and minimizing the energy consumption of the entire network, the algorithm wakes up as few neighbor nodes as possible, to improve the service life of the entire network.

## 2. Network Model

**Figure 1:**
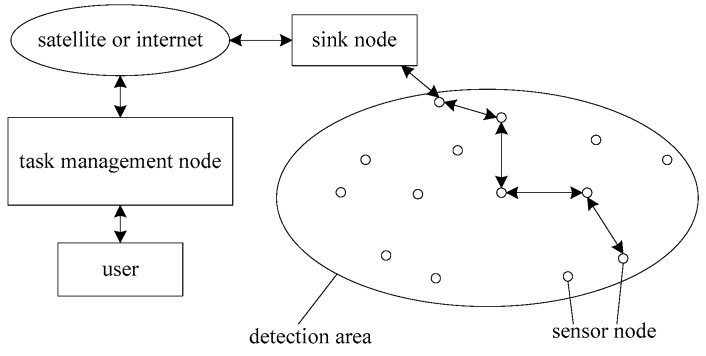
The architecture of WSNs.

The sensor network is a type of wireless network technology that integrates sensor, micromotor systems, and computer technology, and can achieve information acquisition, information processing, and information transmission. The architecture of a typical WSN [10] is shown in Figure 1, including three parts that are sensor nodes, the sink node, and the management node. 

Sensor nodes constitute the hardware basis of the network and the core of the sensor network. They have three functions. First, they complete the information collection as the on-site information collection unit. Second, they transmit the collected field information to other nodes or information processing centers in a certain way. Third, they implement some control functions together with the control module. Additionally, some applications require sensor nodes to perform additional functions, typically including positioning systems, motion or actuators, power regeneration devices, and so on. In the sensor networks, a large number of sensor nodes are randomly scattered near or inside the monitoring area utilizing aircraft broadcast, artificial configuration or rocket ejection, and constitute the network in a self-organization way. The data monitored by the sensor node is transmitted to the sink node by other sensor nodes through wireless single or multiple hop communication, then the sink node transmits it to the task management node through the Internet, mobile communication network, satellite communications, and other means, the collected information is eventually processed by a remote client. Across the entire network, sensor nodes should not only take into account the dual function of terminals and routes in the traditional network, but also store, process and transmit data. The relationship between each sensor node is a kind of collaborative work.

On a general level, connectivity refers to the fact that any two sensor nodes in a sensor network can establish a wireless connection, exchange information, and can communicate with other nodes other than the neighbor nodes through a multi-hop mode, which is also the premise for the normal operation of the whole wireless network [11]. From the perspective of probability theory, this paper studies the relationship between the various parameters related to the connectivity, and constructs the probability analysis model of the node connectivity, and finally presents the networking algorithm called the connectivity and energy efficiency (CEE) algorithm, which can guarantee maximum connectivity probability and reduce the network energy consumption.

## 3. Connectivity and Energy Efficiency Algorithm

A collection of the serial numbers for *N* sensor nodes is represented as M={1,2,3,……,N}. The sensor nodes are distributed uniformly in the detection region [12,13]. According to graph theory, set up $D_{ij}$ as the communication distances between node i and node j. The node communication radius is R. If $D_{ij}\leq R$, a communication relationship can be established between sensor node *i* and node *j*, so there is a communication path between node *i* and node *j*. There are multiple paths *L* existing in the wireless sensor networks, where $0\leq L\leq \frac{N(N-1)}{2}$. If and only if $L=\frac{N(N-1)}{2}$ the WSN is called a fully-connected WSN [14]. 

According to the characteristics of WSN, the number of sensor nodes in the monitoring area is enormous, and the density is high, so the number of sensor nodes is N>>10. We set the node communication radius as *R*, where *R* is generally 10 to 300 m [15], and set the area of the detection area as *S*, where $S>>\pi R^{2}$. Thus, the ratio of the communication area of a node to the area can be obtained, that is, the node’s communication probability $P=\frac{\pi R^{2}}{S}<<0.1$.

### 3.1. The Connectivity via Probability Theory

In our previous research [16] has shown that random deployment of nodes is the most common situation, and the number of the nodes randomly sprinkled by aircraft is subject to binomial distribution in the designated monitoring area. In this paper, we mainly use the method of probability theory, starting from the connectivity *K* to explore the network connectivity, that is to say, the wireless sensor network connectivity *K* also obey the binomial distribution, denoted as *B*(*K*,*P*). Moreover, because the number of nodes N>>10, node’s communication probability P<<0.1. The connectivity *K* of the WSN obeys the Poisson distribution in the monitoring area, and the mathematical expectation is $E_{\xi }=\lambda =NP=N\frac{\pi R^{2}}{S}$.

Sensor node connectivity *K* obey the Poisson distribution, so network connectivity probability $P_{r}(X=k)=\frac{e^{-\lambda }\lambda^{k}}{k!}$, among λ=NP, $P=\frac{\pi R^{2}}{S}$, there is:

(1) $$P_{r}(X=k)=\frac{e^{-NP}{\left(NP\right)}^{k}}{k!}=\frac{{\left(N\frac{\pi R^{2}}{S}\right)}^{k}}{k!}e^{-N\frac{\pi R^{2}}{S}}$$

From Equation (1), *N* is the number of nodes, *R* is the communication radius, *K* is the connectivity and is the connectivity probability value, it can be seen that the number of nodes *N* and the communication radius *R* affect the network connectivity probability value $P_{r}$ when the connectivity is *K*. If the probability of network connectivity increases to *K*, the number of nodes *N* must increase or the communication radius *R* must increase as well. In a sensor network, the probability $P_{r}$ of network connectivity *K* can be improved only when the number of nodes *N* satisfies a certain range [17]. If *N* is lower than the specified range, the information between nodes is unreachable, resulting in the phenomenon if disconnected network. If *N* is higher than the specified range, a certain number of redundant nodes will appear in the network, causing a waste of resources, increasing the burden of network communication and affecting the service life of the network. The problems studied in this paper are how to calculate the nodes *N* and the node communication radius *R*, and how to optimize the probability of network connectivity $P_{r}$.

In order to achieve prospective purpose, let the expected value of network node connectivity be *K*, and hope that the number of neighbor nodes of each node in the network can reach *K* or close to *K*, that is, in the interval [K−a, K+b] (0≤a≤K−1, b≥0), we get the higher $P_{r}$, the better [18]. Then in the interval [K−a, K+b] the accumulated value of $P_{r}$ is:

(2) $$P_{r}(K-a\leq x\leq K+b)=\sum_{x=K-a}^{K+b}\frac{{\left(NP\right)}^{x}}{x!}e^{-(NP)}=\sum_{x=K-a}^{K+b}\frac{{\left(N\frac{\pi R^{2}}{S}\right)}^{x}}{x!}e^{-N\frac{\pi R^{2}}{S}}$$

There contains two variables *N* and *R*, while the monitoring area *S* is a constant. We study the relationship between $P_{r}$ and the node communication radius *R*, the number of nodes *N*. 

#### 3.1.1. The Relationship between the Probability of Network Connectivity $P_{r}$ and the Communication Radius R

Equation (2) on the partial derivative of *R*, obtains:

(3) $$\frac{\partial P_{r}}{\partial R}=\sum_{x=K-a}^{K+b}\frac{2N\pi R}{S}e^{-N\frac{\pi R^{2}}{S}}\left[\frac{{\left(N\frac{\pi R^{2}}{S}\right)}^{x-1}}{(x-1)!}-\frac{{\left(N\frac{\pi R^{2}}{S}\right)}^{x}}{x!}\right]$$

Let $\frac{\partial P_{r}}{\partial R}=0$, so $\frac{{\left(N\frac{\pi R^{2}}{S}\right)}^{K-a-1}}{(K-a-1)!}-\frac{{\left(N\frac{\pi R^{2}}{S}\right)}^{K+b}}{(K+b)!}=0$. Then ${\left(N\frac{\pi R^{2}}{S}\right)}^{a+b+1}=\frac{(K+b)!}{(K-a-1)!}$. Then $N\frac{\pi R^{2}}{S}={\left[\frac{(K+b)!}{(K-a-1)!}\right]}^{\frac{1}{a+b+1}}$. Reduction for $R^{2}=\frac{S}{N\pi }{\left[\frac{(K+b)!}{(K-a-1)!}\right]}^{\frac{1}{a+b+1}}$.

(4) $$R=\sqrt{\frac{S}{N\pi }{\left[\frac{(K+b)!}{(K-a-1)!}\right]}^{\frac{1}{a+b+1}}}$$

As can be seen from above calculation, when the monitoring area *S* and the number of nodes *N* are confirmed, the node communication radius *R* take the result of Equation (4), which makes the network connectivity probability to be the largest in the interval [K−a, K+b]. Equation (4) gives the communication radius *R* for the maximum of network connectivity probability.

#### 3.1.2. The Relationship between the Probability of Network Connectivity $P_{r}$ and the Number of Nodes N

Equation (2) on the partial derivative of *N* obtains:

(5) $$\frac{\partial P_{r}}{\partial N}=\sum_{x=K-a}^{K+b}\frac{\pi R^{2}}{S}e^{-N\frac{\pi R^{2}}{S}}\left[\frac{{\left(N\frac{\pi R^{2}}{S}\right)}^{x-1}}{(x-1)!}-\frac{{\left(N\frac{\pi R^{2}}{S}\right)}^{x}}{x!}\right]$$

Let $\frac{\partial P_{r}}{\partial N}=0$, so $\frac{{\left(N\frac{\pi R^{2}}{S}\right)}^{K-a-1}}{(K-a-1)!}-\frac{{\left(N\frac{\pi R^{2}}{S}\right)}^{K+b}}{(K+b)!}=0$. Then ${\left(N\frac{\pi R^{2}}{S}\right)}^{a+b+1}=\frac{(K+b)!}{(K-a-1)!}$. Then $N=\frac{S}{\pi R^{2}}{\left[\frac{(K+b)!}{(K-a-1)!}\right]}^{\frac{1}{a+b+1}}$.

(6) $$N=\frac{S}{\pi R^{2}}{\left[\frac{(K+b)!}{(K-a-1)!}\right]}^{\frac{1}{a+b+1}}$$

It can be seen from the above calculation, when the monitoring area *S* and the node communication radius *R* are confirmed, the number of nodes *N* take the result of Equation (6), which results in the network connectivity probability being the largest in the interval [K−a, K+b]. Additionally, according to Equation (6) we can obtain the number of nodes *N* of the maximum network connectivity probability.

### 3.2. The New Connectivity and Energy Efficiency Network Algorithm

Two conditions affecting the connectivity of the wireless sensor networks are discussed above, that are the number of nodes *N* and the node communication radius *R*. *N* and *R* are given to meet the conditions that the probability of network node connectivity *K* is the largest [19], as shown in Equations (4) and (6). In addition to these two conditions being necessary for network connectivity, network energy consumption also needs to be taken into account. In a sensor network, too many or too few nodes will affect the overall performance of the network. Too few neighbor nodes will result in poor network connectivity and accessibility, and isolated nodes will appear. On the other hand, if there are too many neighbor nodes, it will not only lead to the redundancy of information and waste resources, but also increase the communication burden of the nodes and reduce the service life of the network. Therefore, a new type of network connectivity and energy efficiency algorithm is proposed in this paper, and it cannot only ensure the network connectivity, but can also effectively reduce the energy consumption caused by the unreasonable working method [18]. In other words, under the condition of guarantee the connectivity *K*, the number of awakened sleep nodes should be reduced as far as possible to achieve the purpose of energy saving. The expected value of connectivity of a wireless sensors network is set as *K*, the closer the number of neighbor nodes of each node is to *K*, the better. That is, on the interval [K−a,K+b](0≤a≤k−1,b≥0), the higher the value of $P_{r}(K-a\leq x\leq K+b)$ is, the better.

According to Equation (4), when the sprinkling area *S* and the number of nodes *N* are known, the communication radius *R* of sensor nodes under the maximum network connectivity probability can be calculated. Given the sprinkling area *S* of the sensor and the communication radius *R* of the sensor node, then, according to Equation (6), the number of sensor nodes *N* under the maximum network connectivity probability can be calculated. Connectivity *K* of wireless sensor network nodes is subject to binomial distribution denoted by B(K,P), among $P=\frac{\pi R^{2}}{S}$, *P* is the probability that the communication coverage area of the node occupies the total area. Within a certain area, if we want to increase the probability of the network connectivity *K*, then the number of sensor nodes *N* should be increased or the communication radius *R* of sensor nodes should be increased. 

The algorithm is a decentralized one in this paper. *K* is denoted as the connectivity of the node, if the distance between any two nodes $D_{i}$ and $D_{j}$ in the wireless sensor network is less than or equal to the communication radius *R* of the nodes, as $D_{ij}\leq R$, so these two nodes are neighbors to each other. If a node has the number of neighbor nodes is *K*, the connectivity of the node is *K*. All nodes are connected in a chain, and nodes of similar positions become neighbors on the chain. Each node receives data from one neighbor, merges its own data, and sends it to another neighbor. This process proceeds along the chain until it reaches the head node of the round, which sends the aggregated results to the base station. Only one head node is specified per turn, and each node, in turn, becomes the head node. We can describe the decentralized CEE algorithm in a procedural way as Algorithm 1.
**Algorithm 1.** Connectivity and Energy Efficiency (CEE) Algorithm.Initialize parameters including K, N, R, and S
**Repeat**Update list of neighbor node $M_{i}$
**If**$M_{i}\cap M_{s}\neq \phi$, **Then**/* Determine which node for waking up by its energy and distance to node i */$W_{i}\leftarrow -\infty$$j_{\mathrm{SEL}}\leftarrow 0$ /* Node selected to wake up */**While**$j\in M_{w}$**Do**$D_{ij}\leftarrow \left|T_{i}-T_{j}\right|V$$w_{ij}\leftarrow \omega_{1}E_{j}+\omega_{2}D_{ij}$**If**$w_{ij}>W_{i}$**Then**$W_{i}\leftarrow w_{ij}$ /* Search for node j with the biggest $W_{i}$ */$j_{\mathrm{SEL}}\leftarrow j$**End If****End While**Wake up the selected node *j*$M_{s}\leftarrow M_{s}-\left\{j_{\mathrm{SEL}}\right\}$$M_{w}\leftarrow M_{w}+\left\{j_{\mathrm{SEL}}\right\}$**End If**$T_{i}\leftarrow T_{i}+\Delta T$ /* Go to next time slot */$N_{i}\leftarrow \mathrm{card}\left(M_{i}\right)$**Until**$N_{i}\geq K$

The new network connectivity and energy efficiency algorithm is described in detail below. Set up the number of sensor nodes in the monitoring area as *N*, their collection is M={1,2,3,……,N}. The number of nodes in working condition is $N_{w}$, its collection is $M_{w}$, then there are $\left(N-N_{w}\right)$ nodes in the sleeping state, its collection is $M_{s}$. The above collections meet $M_{w}\subset M,M_{s}\subset M$, and $M_{w}+M_{s}=M$. For $\forall i\in M_{w}$, the collection is composed of its neighbor nodes as $M_{i}$, moreover the number of elements in the $M_{i}$ is $N_{i}\leq K$. In Step 1, initialize and calculate the parameters.

According to the previous section on the derived formula, calculate the connectivity *K* and the connectivity probability *P_r_* as the output, and the number of nodes *N*, the node communication radius *R*, and the monitoring area *S* are the inputs.

According to Equation (4), when the sprinkling area *S* and the number of node *N* are known, the communication radius *R* of sensor nodes under the maximum network connectivity probability can be calculated.

Given the sprinkling area *S* of the sensor and the communication radius *R* of the sensor node, then, according to Equation (6), the number of sensor nodes *N* under the maximum network connectivity probability can be calculated.

Connectivity *K* of wireless sensor network nodes is subject to binomial distribution, denoted by *B(K,P)*, among $P=\frac{\pi R^{2}}{S}$, *P* is the probability that the communication coverage area of the node occupies the total area. Within a certain area, if we want to increase the probability of the network connectivity *K*, then the number of sensor nodes *N* should be increased or the communication radius *R* of sensor nodes should be increased. In Step 2, update the list of neighbor nodes’ information.

In a particular time $T_{i}$, for any node $i\in M_{w}$, which send a single hop broadcast, establish communication with all of its neighbor nodes $M_{i}$, require them to send their own information to the node *I*, such as the identity of the node, self-state information (waking or sleeping), the sent timestamp $T_{p}$, the residual energy $E_{j}$, and so on. Node *i* in $M_{i}$ receives the related information from all the other nodes, updates the node information list immediately, and then filters which nodes in line meet the requirements of node communication. It is similar to how routers update the routing table in the computer network.

In Step 3, achieve network connectivity *K*.

If the number of neighbor nodes of node *i* reaches *K*, this shows network connectivity can be achieved and the networking ends. 

If the number of working neighbor nodes of node *i* is less than *K*, a certain number of neighbor nodes must be awakened to guarantee the network connectivity reaches *K*. 

If $M_{i}\cap M_{s}\neq \phi$, this indicates that the sleeping neighbor nodes that meet the requirements can be selected to meet the requirements of network connectivity, and Step 4 can be performed at this time. 

If $M_{i}\cap M_{s}=\phi$, this means that all the neighbor nodes of node *i* are waking, in order to meet the requirements of network connectivity, other nodes must be selected again, then we proceed to Step 2. 

In Step 4, wake up selected nodes.

According to Step 2, the neighbor information list of node *i* includes the information of all its neighbor nodes, when the node *i* cannot reach connectivity *K*, it is necessary to wake up the sleeping nodes to meet the requirements. With respect to the kind of nodes that can be awakened to achieve the connectivity and energy saving requirement of the network, [20,21] introduced the method to wake up the sleeping nodes according to the energy efficiency algorithm on the residual energy and relative distance. In this paper, we consider the residual energy denoted as $E_{j}$ and the relative distance $D_{ij}$, also the communication distances difference between node *i* and node j then the $W_{j}$ can be calculated from Equation (7), we can select the node *j* with larger relative value $W_{j}$ of distance and energy to wake up:

(7) $$W_{j}=\omega_{1}\times E_{j}+\omega_{2}\times D_{{}_{ij}}$$

(8) $$D_{ij}=\Delta T_{ij}\times V$$

(9) $$\Delta T_{ij}=\left|T_{i}-T_{j}\right|$$

Among, $E_{j}$ is the residual energy of node j, $\omega_{1}$ and $\omega_{2}$ are the weighting coefficients, $D_{ij}$ is the communication distances difference between node i and node j. $\Delta T_{ij}$ is the time difference of the information transmission between node *i* and node *j*, *V* is the speed of information transmission. Choose the sleeping node with a large value of $W_{j}$ to wake up, and it can achieve the requirements of the network connectivity and energy saving. The basic idea is distance based weighting method, when the target is close to a node, it will stay in the perceived range of the node for a long time, assign a large weight to a node that is closer to the target. 

In Step 5, update the neighbor information list again.

Let $T_{i}=T_{i}+\Delta T$, select the next moment *T_i_*, and return to Step 2. 

Repeat the above five steps until the connectivity of wireless sensor network reaches *K*, and then end the networking. 

When the node of the network reaches the above expected mean value *K*, stop the activity that wakes up the sleeping nodes, that is, the network ends. However, a node may have more neighbors that exceed *K*, and we can still consider the residual energy $E_{j}$ and the relative distance $D_{ij}$. We then calculate $W_{j}$ from Equation (7), and we can select the node *j* with larger relative value $W_{j}$ of the distance and energy as the number of neighbors. In this way, the network energy consumption can be minimized so as to improve the network lifetime significantly. 

## 4. System Simulation 

### 4.1. The Simulation Performance Index

Table 1 shows the main simulation parameters for the network connectivity and energy efficiency algorithm, set up the monitoring area is similar to a circle, set the monitoring radius r=400 m, and set the number of nodes as N=5000>>10. The area of the monitoring area is about $S=\pi r^{2}\approx 5.03\times 10^{5}\;m^{2}$. The communication radius of the node is 30 m as the maximum, so the communication area of the node is $S^{\prime }=\pi R^{2}\approx 2827.43\;m^{2}$. Then, the node’s communication probability $P=\frac{S^{\prime }}{S}\approx 0.0056\ll 0.1$; therefore, the nodes thrown by the aircraft satisfy the Poisson distribution of the mean value λ=NP. 

Up until now, the value range of R of the sensors nodes is higher than 30 m. However, only when the communication radius is greater than twice that of the perceived radius can the connectivity be guaranteed under the condition of certain coverage. However, the detection radius and communication radius of sensor nodes are limited by various aspects, and often coverage nodes can meet the requirements of coverage but cannot guarantee the network connectivity. Then, in a fixed region, only when how many nodes are scattered and what is the connectivity *K* of the nodes, can a wireless sensor network with high reliability, high connectivity probability, and long service life be guaranteed. Due to the cost and difficulty of deploying large-scale sensor networks, the current research work in this paper is carried out in the simulation environment, including the algorithm performance evaluation. For the convenience of analysis, the ideal model is used. The algorithm design uses the connected radius and the circular region, because it simplifies the analysis. The connecting radius of the sensor is different under different sending power. In the case of a certain receiving sensitivity, the relation between the wireless transmitting power *P* and the receiving radius *R* is $P=R^{2}\sim R^{5}$, so *P* could be much larger than $R^{2}$.

Combined with the algorithm for judging connectivity, when calculating coverage, we use the idea of infinitesimal elements to ensure that the network is just connected, then find the minimum communication radius of each node when they are just connected. In terms of implementation, information exchange and maintenance are expensive jobs. Whether it’s centralized computing or distributed computing, there is a large amount of communication overhead, and the overhead is related to the signal power of the nodes and the average node density of the network. In order to adapt to the practical application in the field of wireless sensor network, the model and theory are extended to a larger *R* of about 100 m as our next research item.

**Figure 2:**
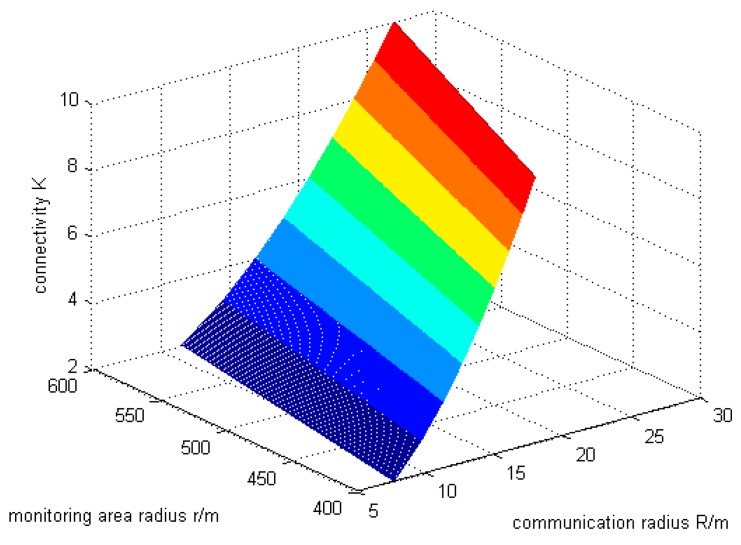
The relationship between the network connectivity and node communication radius.

In the previous section, a new type of network connectivity and energy efficiency algorithm is proposed to minimize the energy consumption while ensuring the maximum probability of network node connectivity *K* in the wireless sensor network. Using the parameters specified above, according to the experimental results, the simulation diagram of the relationship among the network node connectivity *K*, the radius *r* of the monitoring area and the node communication radius *R* is obtained, as shown in Figure 2. 

As can be seen from the Figure 2, when the radius of the monitoring area r=400 m, network connectivity *K* is about 8, node communication radius *R* is about 16 m, and the WSN has strong characteristics of network connectivity [22]. That is to say, when the node communication radius is around 16 m, the entire network can be in the connected state to maintain the normal operation, and the network connectivity expectations *K* is 8, at this time the connectivity probability $P_{r}$ is the greatest.

Since the node communication radius *R* is one of the indicators reflecting the overall network energy consumption, the larger the value of *R* is, the higher the energy consumption of the entire network will be [23]. Figure 2 also shows that a node communication radius *R* of only about 16 can meet the demand for network connectivity, also proving that the new network connectivity and energy efficiency algorithm has the effect of energy saving. The network energy consumption can be effectively reduced while maintaining the desired network connectivity.

The following experiments mainly focus on the information provided by this three-dimensional graphics.

### 4.2. The Simulation Results of the Communication Radius on the Connecting Performance

**Figure 3:**
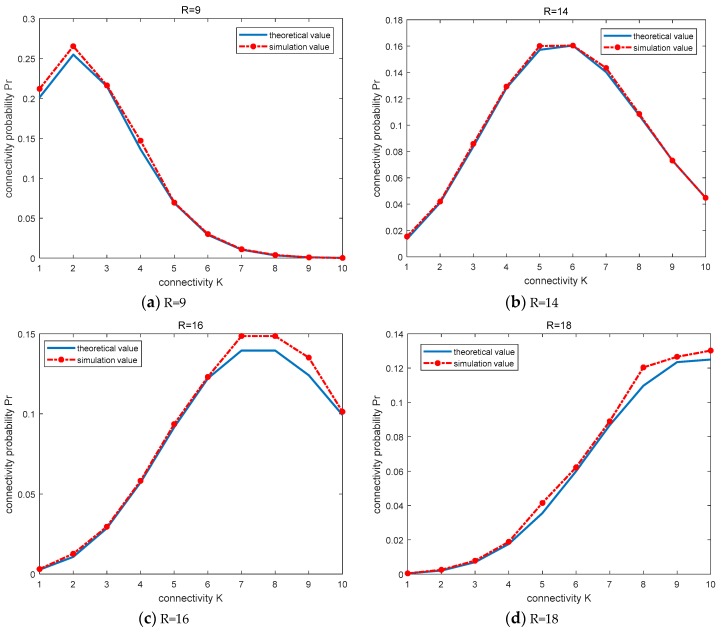
The relationship between the network node connectivity *K* and the connected probability $P_{r}$.

The simulation is carried out in two different conditions, (1) when the node communication radius *R* is determined, the functional relationship between the node connectivity K and the connectivity probability *P_r_*, and (2) when the network node connectivity K=8, the functional relationship between the node communication radius *R* and the connectivity probability $P_{r}$. In order to guarantee the accuracy of the results, every 50 sprinklers were divided into a group, up to 100 groups, a total of 5000 times [22]. This experiment is mainly to determine the statistics of these 100 groups of data, then take any node in each set of experiments, count the number of its neighbor nodes according to the given communication radius *R*. Then we calculate the connectivity probability $P_{r}$, where the range of connectivity *K* is 0~10 [24], the communication radius *R* is, in turn, *R* = 9, *R* = 14, *R* = 16, and *R* = 18. The simulations are shown in Figure 3. 

.

Figure 3 shows the function curve of the network node connectivity *K* and the connectivity probability *P_r_* when the node communication radius is *R* = 9, *R* = 14, *R* = 16, and *R* = 18, respectively. Among them, the simulation curve is drawn based on the results of 100 groups of experiments, and the theoretical curve is derived from the calculation formula of Poisson distribution [25]. As can be seen from Figure 3, the simulation curve is close to the theoretical curve, that is to say, the error is relatively small. We can prove that the number of sensor nodes randomly sprinkled in the monitoring area can be approximately regarded as the Poisson distribution. Thus, the network node connectivity *K* also conforms to the principle of Poisson distribution.

In addition, we analyze the above four Figures one by one, in Figure 3a, when the node communication radius *R* = 9, and the connectivity *K* = 2, the probability is the largest. In Figure 3b, when the node communication radius *R* = 14, and the connectivity *K* = 6, the probability is the largest. In Figure 3c, when the node communication radius *R* = 16, and the connectivity *K* = 7 or *K* = 8, the probability is the largest, at this point the network is firmly connected. Figure 3d when the node communication radius *R* = 18, the connectivity probability is the largest, and the connectivity *K* has exceeded the given range. It can be concluded that, when the node communication radius *R* is larger, the expected network connectivity *K* is greater. In other words, in order for the greater network connectivity *K*, the node communication radius *R* must be larger [26]. According to this discipline combined with the new network connectivity and energy efficiency algorithm, the algorithm chooses the optimal sleep nodes to wake up, to make the network to the desired connectivity, at the same time, the energy consumption can be reduced to the minimum.

### 4.3. The Simulation Results of the Connectivity on the Connectivity Probability

**Figure 4:**
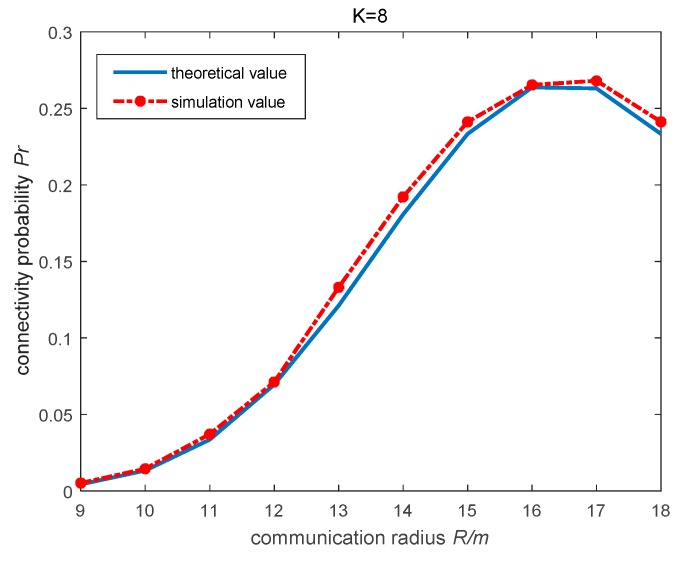
The relationship between the node communication radius and the connected probability diagram $P_{r}$ when the K = 8.

According to Figure 3, we know that when the connectivity *K* is about 8, communication radius *R* is about 16, the wireless sensor network is a strongly connected network. Thus, when the connectivity *K* = 8, and the node communication radius *R* takes different values, the theoretical values obtained from Equation (2) are compared with the experimental results. The node communication radius *R* is 9~18, and both *a* and *b* are equal to 1. When K=8, the relationship between the node communication radius *R* and connectivity probability *P_r_* is shown in Figure 4.

As can be seen from the Figure 4, the simulation curve is very approximate with the theoretical curve, so in the actual situation can use Equation (2) to compute the maximum of the connectivity probability. When and only when the communication radius *R* = 16, the network connectivity *K* = 8, the wireless sensor network achieve the highest connectivity probability. This proves the conclusion from the fact that Figure 3 reflects, when the network connectivity K=8 and node communication radius R=16, the wireless sensor network can perform robust connectivity. Moreover, if the node communication radius *R* continues to increase, such as R=18, the network connectivity probability $P_{r}$ fell to 0.23. It cannot guarantee the network connectivity to be maximized and will increase the network energy consumption as well [27], therefore, reducing the service life of the network. 

## 5. Conclusions

This paper researches the connectivity of WSNs, combines the probability theory with the new network connectivity and energy efficiency algorithm, the principle of updating routing table by a router in the computer network is applied to the WSNs, which explains the information exchange between neighbor nodes, such as updating the list of neighbor information. The innovation of the paper is to choose which type of sleeping node to wake up according to the residual energy and the relative distance of the nodes. In order to maximize the network connectivity and the connectivity probability, and minimize the energy consumption of the entire network as much as possible, the algorithm wakes up as few neighbor nodes as possible, to improve the service life of the whole network. According to the random deployment of sensor nodes satisfying a Poisson distribution, the simulation results show the network reaches the maximum network connectivity and connectivity probability, and the expression and the value of the node communication radius *R* and the number of nodes *N* are given. According to the parameters provided by the strongly-connected network, under different node communication radius *R*, the relationship between the node connectivity *K* and the connectivity probability *P_r_* is presented. When the connectivity *K* = 8, according to the relationship between the node communication radius *R* and the connectivity probability $P_{r}$, the algorithm maximizes the connectivity and minimizes the energy consumption in the WSNs. 

## Figures and Tables

**Table 1:** Simulation parameters for the network connectivity and energy efficiency algorithm.

Parameter	Values
Monitoring radius (*r*)	400 m
N	5000
The communication radius of the node (*R*)	30 m
